# Preliminary Study of Vibrotactile Feedback during Home-Based Balance and Coordination Training in Individuals with Cerebellar Ataxia

**DOI:** 10.3390/s22093512

**Published:** 2022-05-05

**Authors:** Safa Jabri, David D. Bushart, Catherine Kinnaird, Tian Bao, Angel Bu, Vikram G. Shakkottai, Kathleen H. Sienko

**Affiliations:** 1 Department of Mechanical Engineering, University of Michigan, Ann Arbor, MI 48109, USA; safajb@umich.edu (S.J.); catherinekinnaird@gmail.com (C.K.); baotian@umich.edu (T.B.); angelbu@mit.edu (A.B.); 2 Department of Neurology, University of Michigan Medical School, Ann Arbor, MI 48109, USA; david.bushart@osumc.edu; 3 Department of Molecular and Integrative Physiology, University of Michigan Medical School, Ann Arbor, MI 48109, USA; 4 The Ohio State University College of Medicine, Ohio State University, Columbus, OH 43210, USA; 5 Department of Neurology, University of Texas Southwestern Medical Center, Dallas, TX 75390, USA

**Keywords:** sensory augmentation, balance rehabilitation, telerehabilitation, wearable sensors, cerebellar ataxia

## Abstract

Intensive balance and coordination training is the mainstay of treatment for symptoms of impaired balance and mobility in individuals with hereditary cerebellar ataxia. In this study, we compared the effects of home-based balance and coordination training with and without vibrotactile SA for individuals with hereditary cerebellar ataxia. Ten participants (five males, five females; 47  ±  12 years) with inherited forms of cerebellar ataxia were recruited to participate in a 12-week crossover study during which they completed two six-week blocks of balance and coordination training with and without vibrotactile SA. Participants were instructed to perform balance and coordination exercises five times per week using smartphone balance trainers that provided written, graphic, and video guidance and measured trunk sway. The pre-, per-, and post-training performance were assessed using the Scale for the Assessment and Rating of Ataxia (SARA), SARA_posture&gait_ sub-scores, Dynamic Gait Index, modified Clinical Test of Sensory Interaction in Balance, Timed Up and Go performed with and without a cup of water, and multiple kinematic measures of postural sway measured with a single inertial measurement unit placed on the participants’ trunks. To explore the effects of training with and without vibrotactile SA, we compared the changes in performance achieved after participants completed each six-week block of training. Among the seven participants who completed both blocks of training, the change in the SARA scores and SARA_posture&gait_ sub-scores following training with vibrotactile SA was not significantly different from the change achieved following training without SA (p>0.05). However, a trend toward improved SARA scores and SARA_posture&gait_ sub-scores was observed following training with vibrotactile SA; compared to their pre-vibrotacile SA training scores, participants significantly improved their SARA scores (mean=−1.21,  p=0.02) and SARA_posture&gait_ sub-scores (mean=−1.00,  p=0.01). In contrast, no significant changes in SARA scores and SARA_posture&gait_ sub-scores were observed following the six weeks of training without SA compared to their pre-training scores immediately preceding the training block without vibrotactile SA (p>0.05). No significant changes in trunk kinematic sway parameters were observed as a result of training (p>0.05). Based on the findings from this preliminary study, balance and coordination training improved the participants’ motor performance, as captured through the SARA. Vibrotactile SA may be a beneficial addition to training regimens for individuals with hereditary cerebellar ataxia, but additional research with larger sample sizes is needed to assess the significance and generalizability of these findings.

## 1. Introduction

Hereditary cerebellar ataxia are a group of neurological disorders characterized by the degeneration of the cerebellum and its associated pathways. The cerebellum is primarily responsible for adaptation and motor learning. Individuals with degenerative cerebellar ataxia have progressive impairments in motor coordination [1,2] and sensorimotor processing [3], resulting in unsteadiness in their gait and posture. The symptoms include uncoordinated multi-joint limb movements, as well as impaired postural responses and an increased risk of falls, frequently leading to wheelchair confinement [4]. These balance and coordination deficits are typically reflected through increased spatial variability of movements [4], abnormal postural sway [5,6], and increased variability in step length, step width, and gait velocity [7,8]. These symptoms stem from the interaction of multiple factors including: motor deficits in intra-limb coordination [9], oculomotor deficits in visually guided locomotor tasks [10], sensorimotor processing deficits resulting in incorrect balance response scaling to visual perturbation [11], and deficient sensorimotor movement prediction [12].

Physical therapy with balance and coordination training is currently the mainstay of treatment for impaired balance and mobility in individuals with hereditary cerebellar ataxia [13,14]. Balance and coordination training aims to leverage the central nervous system’s ability to learn new strategies to improve motor function through motor learning and adaptation [15], and to reweight intact sensory inputs in the event of sensory deficits [16,17,18,19]. Physical therapy that includes balance and coordination tasks has been shown, in small observational studies, to lead to improvements in motor function immediately after training among individuals with hereditary cerebellar ataxia. Large, prospective randomized controlled studies have not been yet performed to show the long-term, sustained benefit of physical therapy as a therapeutic intervention [4,14,20].

Small, preliminary studies suggest that balance and coordination rehabilitation programs lead to improvements in measures of motor performance in participants with hereditary cerebellar ataxia [13,20,21,22,23,24,25,26,27]. For example, a longitudinal observational study by Seco et al. [28] examined the effects of a long-term rehabilitation program with a focus on functional, balance, and coordination training for participants with Friedreich’s ataxia (a type of autosomal recessive hereditary cerebellar ataxia) over five years. In this study, an intervention group received rehabilitative treatment three times per week for 60 min under the supervision of a physical therapist, while a control group did not participate in the rehabilitation program. The findings from this study indicated that the intervention group was able to maintain their International Cooperative Ataxia Rating Scale (ICARS) [29] scores at the end of the five-year training period and two years after the completion of the training while the control group’s scores progressively worsened [28]. These findings suggest the importance of continued training to stabilize the progression of hereditary cerebellar ataxia. However, long-term access to frequent in-clinic physical therapy may be a challenge, making home-based training an important alternative to consider.

A study by Ilg et al. [21] examined the effects of a four-week home-based coordinative training program. The findings from this study indicated a significant decrease in the Scale for the Assessment and Rating of Ataxia (SARA) scores, a widely used rating scale for motor impairment in participants with hereditary cerebellar ataxia, after the four-week training program. Participants were instructed to continue performing the training following the completion of the four-week program for one year; the SARA scores remained significantly lower than the baseline scores despite the progressive nature of degenerative hereditary cerebellar ataxia following the year of home-based training [21]. In addition, a study by Keller et al. [26] examining the effects of six weeks of home-based static and dynamic balance training found improvements in some outcome measures, including walking speed, but no significant changes in ICARS scores. Each participant in this study received a personalized exercise program developed by a physical therapist based on an initial assessment. The participants were asked to complete training sessions four to six days per week for a minimum of 20 min per session. The findings from this study indicated that increased levels of challenge during home-based training resulted in greater improvements in performance [26]. 

Challenges associated with home-based balance and coordination training include the lack of supervision and feedback. Prior studies have found that supervised training led to improved outcomes compared to home-based training in participants with vestibular disorders [30] and older adults [31,32]. Findings by Barbuto et al. [33] suggest that supervised training may also be more beneficial than unsupervised training for participants with hereditary cerebellar ataxia. Sensory augmentation (SA) is a technique of augmenting compromised sensory information [34,35] that could be used in the context of home-based balance and coordination training to provide exercisers with feedback on their performance. While balance and coordination impairments in individuals with hereditary cerebellar ataxia result from multi-factorial motor and sensorimotor control and integration issues, SA has the potential to address some of these deficits by providing enhanced afferent information to support sensory feedback. A recent study by Zimmet et al. [36] reported that participants with cerebellar ataxia were able to use altered (phase-advanced) visual feedback to improve control in a reaching task, indicating that this population could leverage SA to improve motor performance.

SA devices for balance and coordination training applications typically include sensors and a feedback display to provide body-motion cues [37]. Wearable sensors, such as inertial measurement units (IMUs) [38,39,40], pressure insoles [40] and electromyography (EMG) sensors [41], have previously been used in balance training studies. IMUs are particularly well suited for sensory-augmented balance and coordination training, since they are widely integrated into wearable or portable wireless devices, such as smartwatches and phones. Regardless of the specific type of feedback modality (vibrotactile feedback [42], surface electrode stimulation of the vestibular nerve [43], electric currents applied to the tongue [44,45,46], auditory [47,48], visual [49], or multimodal feedback [50]), participants with sensory disabilities (e.g., vestibular disabilities [42,51], peripheral neuropathy [52], and motor disabilities (e.g., Parkinson’s disease [53,54,55]) have used SA cues to make postural and gait-related corrections.

To date, a limited number of studies have investigated the effects of balance and coordination training with SA among individuals with hereditary cerebellar ataxia. In a preliminary study by Čakrt et al. [44], seven participants with degenerative cerebellar ataxia performed an intensive two-week, 20-session balance rehabilitation program, during which they received tongue electrotactile SA. Balance performance was assessed by measuring head movements in the anterior/posterior (AP) and medial/lateral (ML) directions using an accelerometer at the beginning, the end, and four weeks after the completion of the two-week program. This training program was found to significantly reduce the mean velocity of the center of pressure (CoP) and 95% confidence elliptical area of CoP sway during closed-eyes standing tasks [44]. While these findings indicate that the participants benefited from the training by improving their balance performance, the lack of a control group in this study did not allow the effects of training alone versus training with tongue electrotactile SA to be determined. Additionally, a recent pilot study by Therrien et al. [56] examined the effect of augmenting training with binary reinforcement feedback through an auditory cue to improve reaching task performance for participants with cerebellar ataxia. The results from this study showed that the participants reduced their path length during the reaching tasks compared to repeated training without auditory feedback [56]. Neither study, however, used any clinically relevant cerebellar ataxia rating scale to determine whether the observed improvements were applicable to function. While these recent studies provide preliminary evidence of improvements in outcome measures following training with SA, further investigation using clinically relevant, cerebellar ataxia-specific rating scales is needed to assess the effects of SA on unsupervised home-based balance and coordination training for individuals with hereditary cerebellar ataxia.

The goal of this preliminary study was to examine the effects of home-based balance and coordination training with and without vibrotactile SA on postural stability, gait, and coordination in individuals with hereditary cerebellar ataxia.

## 2. Methods

### 2.1. Study Cohort

Ten individuals diagnosed with hereditary cerebellar ataxia (Table 1) were recruited to participate in a 12-week home-based balance and coordination training program. All participants were able to stand for at least 10 s and walk 10 m with only intermittent support and demonstrated intact cognition (Mini-Mental-State Examination score ≥ 24/30 [57]). Participants were excluded if they were diagnosed with any other disorder that may have affected balance or movement beyond ataxia, or if they had a severe vision or hearing impairment that was not correctable by using glasses, contact lenses or hearing aids. All participants provided written informed consent, and the study was conducted in accordance with the Declaration of Helsinki. The study was reviewed and approved by the University of Michigan Institutional Review Board (HUM00116756).

**Figure 1:**
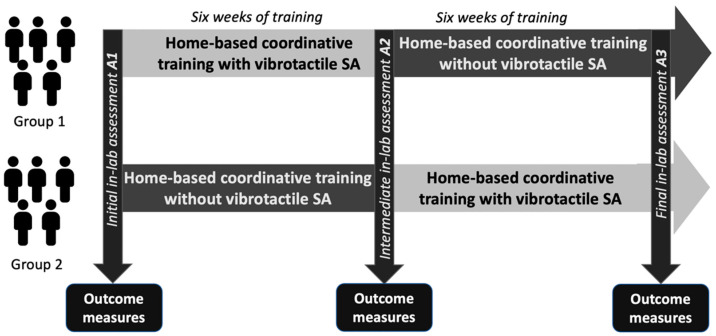
Schematic illustrating the 2 × 2 crossover experimental design used in this study. Participants followed a home-based balance and coordination training program for 12 weeks split into two six-week blocks. Following an initial laboratory-based performance assessment (A1), participants were split into two groups. **Group 1** performed home-based training with vibrotactile SA for the first six weeks then trained without vibrotactile SA for the following six weeks. **Group 2** performed home-based training without vibrotactile SA for the first six weeks then trained with vibrotactile SA for the following six weeks. After each block of six weeks, participants’ balance and coordination were assessed halfway through (A2) and at the end (A3) of the 12-week intervention.

In this study, we used a 2 × 2 crossover experimental design such that each participant served as their own control to maximize statistical power given the limited sample size. We did not include a washout period because an appropriate length for a washout period for this population and intervention had not been established; prior work has shown evidence of sustained training effects for up to a year with continued home-based training following four weeks of intensive balance and coordination training [21]. Participants completed three separate days of laboratory-based balance and coordination assessments immediately before (initial assessment A1), halfway through (intermediate assessment A2), and immediately after (final assessment A3) 12 weeks of home-based balance and coordination training. The 12-week home-based training protocol was split into two six-week blocks (Figure 1) and participants were randomly assigned to one of two groups. After the initial assessment A1, Group 1 participants (n = 5, 46 ± 13 years., 3 males/2 females) performed the first six weeks of their home-based training using vibrotactile SA. Group 2 participants (n = 5, mean 48 ± 13 years, 2 males/3 females), on the other hand, performed home-based training without vibrotactile SA for the first six weeks. Participants’ balance and coordination were reassessed after the completion of the first six weeks of training (intermediate assessment A2) in the laboratory. After the completion of the intermediate assessment A2, Group 1 participants performed the next six weeks of home-based training without vibrotactile SA, while Group 2 participants completed the training protocol with vibrotactile SA. Following completion of the 12-week training protocol, participants’ post-training balance and coordination were reassessed again in the laboratory (final assessment A3).

### 2.2. Home-Based Balance and Coordination Training

**Figure 2:**
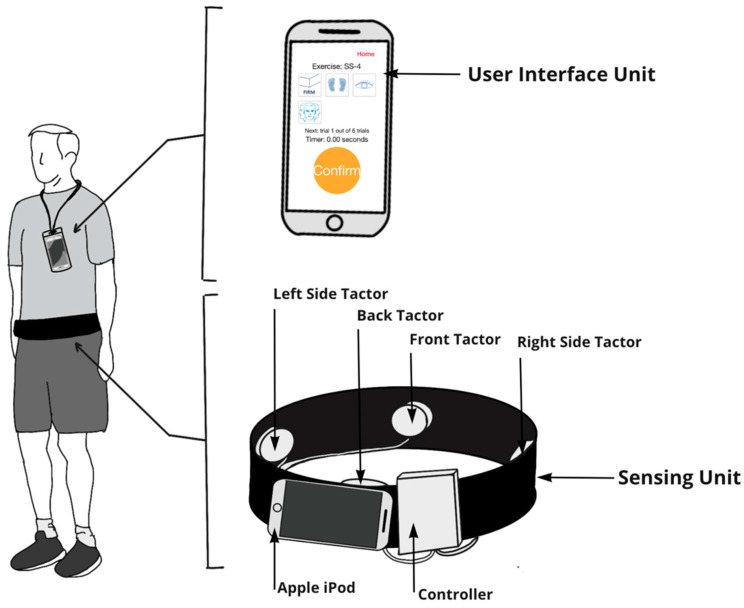
The smartphone-based balance trainer included a user interface unit (Apple iPod) and sensing unit (elastic band with a sensing Apple iPod and four tactors to provide vibrotactile SA). Participants were instructed to wear the user interface unit on a lanyard while performing exercises. The user interface unit allowed participants to select exercises and acted as a timer that instructed participants to start and stop exercises. The sensing unit used (1) the tri-axial gyroscopes embedded in the Apple iPod to measure ML and AP angular velocities, (2) both the tri-axial accelerometers and gyroscopes embedded in the Apple iPod to estimate tilt with respect to gravitational acceleration, and (3) an audio signal to trigger the four tactors to provide vibrotactile feedback. Participants were instructed to make a postural correction in the opposite direction (“move away from the vibration”) when they perceived a vibration.

All participants wore a smartphone balance trainer during home-based balance and coordination training sessions regardless of whether they received vibrotactile SA. The balance trainer, described in detail in Bao et al. [38], comprised an Apple iPod (sixth-generation iPod touch, 2015) (sensing unit) and four tactor buds mounted on an elastic belt, as well as a handheld Apple iPod (sixth-generation iPod touch, 2015) (user interface unit). The sensing unit was attached to the participant’s body around their lower back, approximately at the level corresponding to the L4/L5 spinal segment, to measure trunk sway. The four tactors provided directional vibrotactile cues aligned with the navel, lumbar spine, and right and left sides of the trunk (Figure 2).

**Figure 3:**
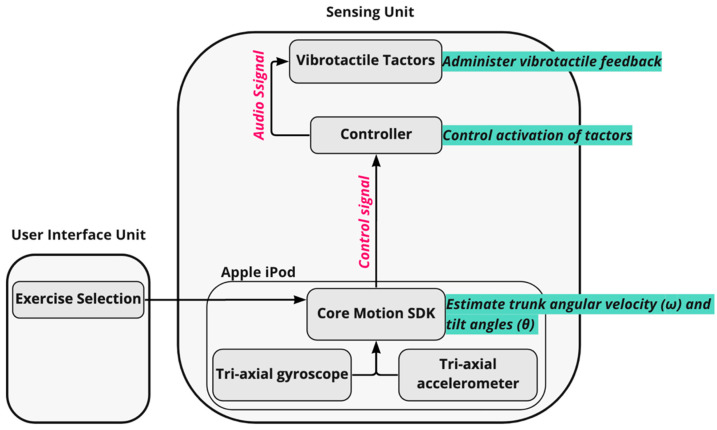
Schematic demonstrating the architecture of the vibrotactile feedback algorithm used in the smartphone-based balance trainer. For each exercise, tilt angles and angular velocities extracted from the Core Motion SDK were used to determine when the vibrating actuators (tactors) should be activated. A control signal (trunk tilt plus one half of the angular rate of tilt [42]) was used for static standing, compliant standing, and arm-raise exercises. However, the control signal only considered trunk tilt for the weight shifting exercises. When a participant’s control signal exceeded a pre-defined threshold in a particular direction (Table 2), the tactor bud in the direction of movement was activated via an audio signal to provide a vibrotactile cue to the participant.

The sensing unit measured trunk angular velocities via tri-axial gyroscopes embedded in the waist-mounted Apple iPod (50 Hz sampling frequency). Tilt angles (angular displacements) were estimated based on gravitational outputs (Class CoreMotion, Apple Inc.) following methods described by Lee et al. [51] in four directions: anterior–posterior (AP) and medio-lateral (ML). The tilt angles and angular velocities were used to determine when vibrotactile SA should be administered (Figure 3). A control signal corresponding to the trunk tilt plus half the angular rate of tilt was used for static standing, compliant standing, and arm-raise exercises [42]. The control signal only considered trunk tilt for weight-shifting exercises. When a participant’s control signal exceeded a pre-defined threshold (Table 2) in a particular direction [42], the tactor bud that most closely aligned with the direction of tilt was activated to provide a vibrotactile cue to the participant. Thresholds to trigger vibrotactile SA were informed by a previously published study [39] and expert input from a physical therapist. Participants were instructed to make a postural correction in the opposite direction (“move away from the vibration”) when they perceived a vibration.

Participants were instructed to perform the prescribed balance and coordination exercises five times per week for 12 weeks (60 sessions in total). Each session lasted approximately 30 min and included exercises from five different categories (Table 2).

Vibrotactile SA was only provided for static standing, standing on a compliant surface, arm-raises, and weight-shifting exercises. Vibrotactile SA was not provided during gait exercises since pre-defined thresholds did not exist for individuals with hereditary cerebellar ataxia and non-intended, less natural gait patterns were observed when SA was used with gait exercise in prior studies [34].

**Figure 4:**
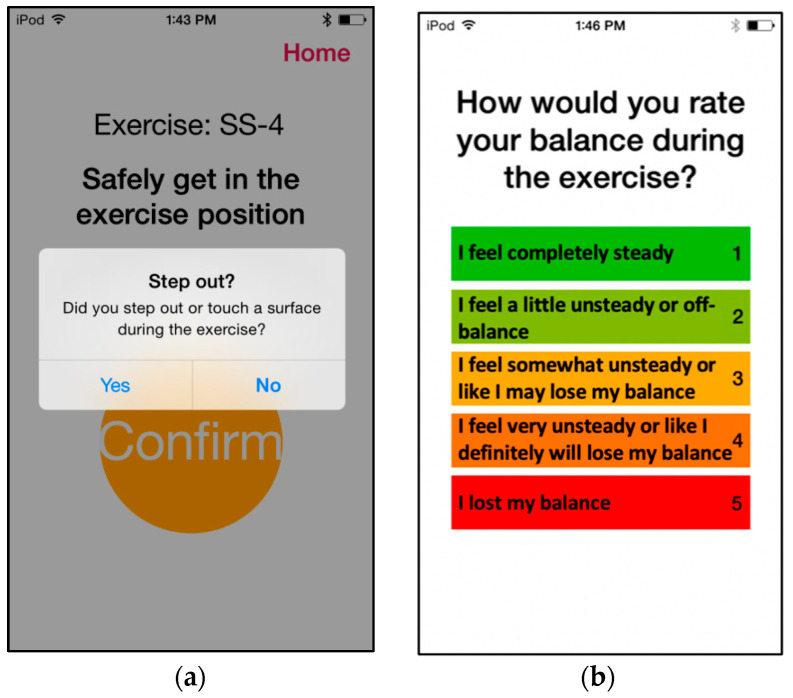
Screenshots showcasing the prompts participants responded to during home-based balance and coordination training sessions. After each repetition of a home-based exercise, participants were prompted (**a**) to report whether they stepped out during the exercise, and (**b**) to indicate a self-rating on the VAS 1–5 scale.

Participants performed six repetitions of six unique exercises per session: one from each of the first four categories and two from the fifth (gait) category. Each exercise was performed for 30 s (except weight-shifting exercises, where the repetition stopped after participants maintained the target positions for five seconds on each side). For sessions involving vibrotactile SA, vibrotactile cues were provided during four (randomly selected) out of the six repetitions per exercise, as prior research reported that a reduced frequency of feedback enhanced motor learning [58,59]. Upon completing each repetition, participants were prompted to log any step-outs that occurred via the iPod user interface. A participant was considered to have stepped out if they had to take a step to regain balance, touch a wall or chair for support, or open their eyes (on tasks for which they were asked to close their eyes). After six repetitions, participants rated their perceived stability on a visual analog scale (VAS) of 1–5 (Figure 4). The step-out and self-rating data were automatically uploaded to a secure cloud server and then sent to a physical therapist (blinded to participant and group designation), who selected customized exercises for each participant on a weekly basis using their clinical judgment and an exercise progression framework modified from previous work [38,60]. The initial exercise assignment was determined during an initial home visit by the study team. The physical therapist assignment aimed to keep participants training at a moderate level of challenge equivalent to a score of 3 on the VAS. Lower VAS self-rating scores with no step-outs resulted in more challenging exercises being assigned, and higher VAS self-rating scores with multiple step-outs resulted in less challenging exercises being assigned until a moderate level of challenge was achieved. In addition, participants completed a weekly activity log to report any pain that limited movement, falls, changes in medication, and injuries.

### 2.3. In-Laboratory Assessments

Initial (A1), intermediate (A2), and final (A3) balance and coordination assessments were performed within a laboratory setting to assess performance prior to starting the home-based training protocol and following each six-week block of training (Figure 1).

The primary clinical outcome measure was the Scale for the Assessment and Rating of Ataxia (SARA) score. The SARA is a clinically validated 0–40 rating scale specifically designed to capture disability that results from cerebellar dysfunction [61]. The SARA protocol includes one gait component, one stance component, one sitting component, one speech component, and four limb-kinetic components. Changes in SARA scores have been used in several recent studies involving participants with hereditary cerebellar ataxia to assess the effects of interventions on balance, gait, and coordination performance [13,49,62]. A lower SARA score represents a lower level of cerebellar impairment. The SARA_posture&gait_ sub-score, a sum of the gait, stance, and sitting components of the SARA [13,63], was also analyzed to capture changes in performance. An experienced neurologist on the study team (V.G.S.) blind to the participants’ group assignments provided SARA ratings after viewing videos of participants performing the SARA.

Additional clinical outcome measures included: modified Clinical Test of Sensory Interaction in Balance (mCTSIB) [64], measuring static postural stability; Dynamic Gait Index (DGI) [65], measuring balance during walking tasks; Timed Up and Go performed with and without a cup of water (TUG [66] and TUG-motor [67]), measuring dynamic stability during functional tasks; and Five-Times Sit-to-Stand Test (5xSST) [68], measuring lower-body strength and transitional movement strategies.

In addition to laboratory-based clinical outcome measures, a single wearable inertial measurement unit (IMU) (MTx, Xsens, Netherlands) was placed on participants’ lower backs, approximately at the level corresponding to the L4/L5 spinal segment, a commonly used IMU placement to assess postural stability during standing [69], to measure their trunk sway (100 Hz sampling frequency) while they performed the mCTSIB test outlined in Table 3.

Multiple kinematic features were calculated from the IMU data, as outlined in Table 4. Root-mean-square (RMS) sway measures in the ML and AP directions were used to capture variance of sway and sway velocity in the time domain [70]. The ellipse area and path length of sway are composite measures that take into account AP and ML sway in two-dimensional space [71]. Ellipse area captures the amplitude of the overall displacement of the participant from their initial position over the course of the 30-second exercise, and path length captures the “angular distance” traveled within that displacement. Lower RMS sway, path length, and ellipse area values are associated with increased postural stability [42,48]. Higher RMS Sway Velocities can indicate increased sway amplitude or sway frequency [72].

### 2.4. Statistical Analysis

All statistical analyses were performed using R-studio (the R Project for Statistical Computing [73]) and significance of results was determined with α = 0.05. Due to the exploratory nature of this study, multiple comparisons were conducted using a variety of outcome measures. We did not perform corrections (such as the Bonferroni correction) to the α value, as such conservative corrections are not recommended in exploratory analyses [74].

To assess the effect of each block of training, we computed a two-way analysis of variance (ANOVA) for each of the clinical outcome measures and the kinematic data collected during the laboratory-based assessments (A1, A2, and A3). In addition, a two-way ANOVA was used to assess the changes in outcome measures at the start of each six-week block of intervention.

Performance was assessed based on each of the clinical outcome measures (SARA, SARA_posture&gait_, TUG, TUG-m, 5xSST, mCTSIB, and DGI), as well as the kinematic features extracted for each of the four exercises performed in the mCTSIB (Table 3).

#### 2.4.1. Effects of 12 Weeks of Training

To assess the overall effect of 12 weeks of training, we considered the effect of $Training_{overall}$ (two levels: $pre_{training}$ and $post_{training}$ corresponding to the initial A1 and final A3 assessments) and the effect group assignment (two levels: Group 1 and Group 2), as well as their interaction (Equation (1)).

(1) $$outcome\;measure\;\sim \;Training_{overall}\times Group\;$$

#### 2.4.2. Effects of Six Weeks of Training with and without Vibrotactile SA

To first assess the effects of each block of training on participants’ performance, we computed a two-way ANOVA for each of the six-week blocks of training. We considered the effect of $Training_{intervention}$ (two levels: $pre_{+SA}$ and $post_{+SA}$ for the six weeks of training with vibrotactile SA, and $pre_{-SA}$ and $post_{-SA}$ for the six weeks of training without vibrotactile SA) and the effect of group assignment (two levels: Group 1 and Group 2) (Equation (2)). The $pre_{+SA}$ level corresponded to the A1 assessment for Group 1 and to the A2 assessment for Group 2. Similarly, the $post_{+SA}$ level corresponded to the A2 assessment for Group 1 and to the A3 assessment for Group 2. Levels for training without SA followed the same pattern, i.e., $pre_{-SA}$ corresponded to the A2 assessment for Group 1 and A1 assessment for Group 2, and $post_{-SA}$ corresponded to A3 assessment for Group 1 and A2 assessment for Group 2 (Figure 1).

(2) $$outcome\;measure\;\sim \;Training_{intervention}\times Group$$

#### 2.4.3. Comparison of the Effects of Training with versus without Vibrotactile SA

In addition, we assessed whether training with vibrotactile SA had an effect on the outcomes of training. We computed a two-way ANOVA to examine the effects of the type of intervention received (two levels: block of training without vibrotactile SA, block of training with vibrotactile SA) and group assignment (two levels: Group 1 and Group 2) on the change in outcome measures achieved after each block of training (Equation (3)).

(3) outcome measure ~ Intervention×Group

## 3. Results

The participants’ initial, intermediate, and final assessment scores for the clinical outcome measures are shown in Table 5. Among the ten recruited study participants, one (Participant 4) withdrew due to an unrelated orthopedic injury before completing the first six weeks of training and was therefore not included in Table 5, and two others (Participants 5 and 7) withdrew after the intermediate assessment, A2, due to the intensity of the training schedule. Seven participants completed the 12-week training protocol. If a participant was missing an outcome measurement, the participant was excluded from the analysis performed for that particular outcome measure.

### 3.1. Analysis of Clinical Outcome Measures

Table 6 summarizes the clinical outcome measure scores at the beginning of each six-week block, as well as the change in scores observed after each six-week block, and after the 12-week protocol. The baseline values for each of the two six-week training blocks (baseline values without vibrotactile SA for Groups 1 and 2 corresponded to assessments A2 and A1, respectively; baseline values with vibrotactile SA for Groups 1 and 2 corresponded to assessments A1 and A2, respectively) were not significantly different for any of the clinical outcome measures.

#### 3.1.1. Effects of 12 Weeks of Training

**Figure 5:**
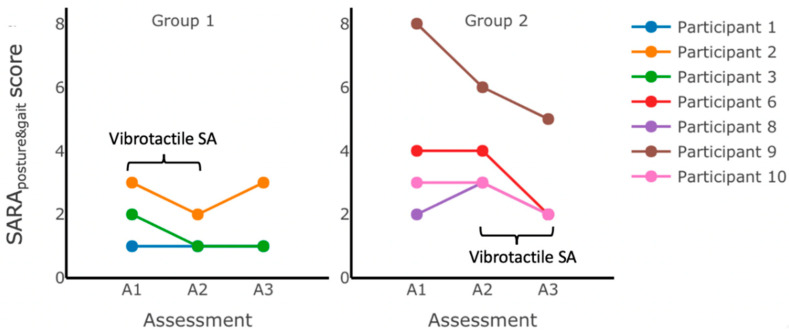
SARA_posture&gait_ scores measured during assessments performed in the laboratory immediately before (initial assessment A1), halfway through (intermediate assessment A2), and immediately after (final assessment A3) home-based balance and coordination training for each group. No statistically significant changes in the SARA_posture&gait_ scores were observed after 12 weeks of training (between A1 and A3), but all participants’ scores improved (Participants 3, 6, 9, and 10) or remained the same as baseline at assessment A1 (Participants 1, 2, and 8). Group 1 received vibrotactile SA during the first six weeks of training (between A1 and A2). Group 2 received vibrotactile SA during the second six weeks of training (between A2 and A3).

We examined the overall effects of 12 weeks of home-based balance and coordination training on participants’ performance using the clinical outcome measures (SARA_posture&gait_, SARA, TUG, TUG-m, 5xSST, mCTSIB, and DGI). The results of this analysis indicated no statistically significant main effect of training. No statistically significant differences (*p* ≥ 0.05) were found when comparing the pre- and post-training scores for any of the clinical outcome measures (Table 6). Although decreasing trends in the SARA (mean (post−pre)=−1.21,SD=1.73, F(1,5)=2.84, p=0.15) and SARA_posture&gait_ scores (mean (post−pre)=−0.71, SD=1.11, F(1,5)=2.29, p=0.19) were observed throughout the 12 weeks of training, the results did not achieve statistical significance (Figure 5). Notably, no decline in performance was detected on any of the clinical outcome measures, and all the measures trended toward improvements (i.e., decreased SARA, TUG, TUG-m, and 5xSST; increased mCTSIB and DGI). No significant main effects of group assignment or interaction were detected.

#### 3.1.2. Effects of Six Weeks of Training without Vibrotactile SA

**Figure 6:**
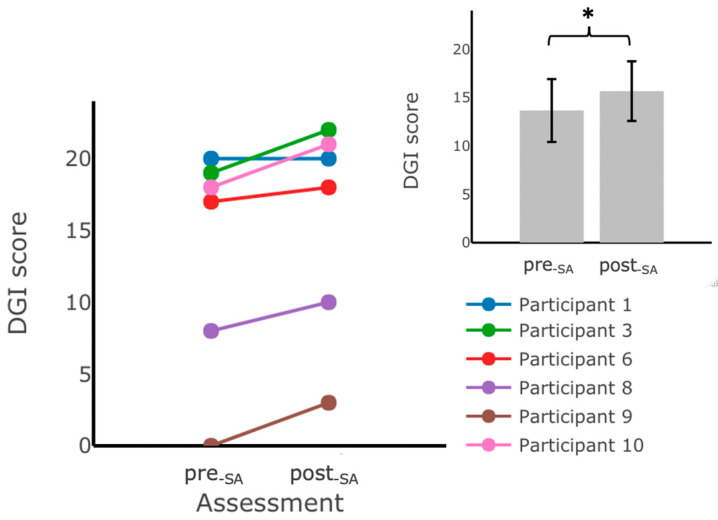
DGI scores measured during assessments performed in the laboratory before ($pre_{-SA}$) and after training at home without vibrotactile SA ($post_{-SA}$). Participants scored significantly higher on the DGI assessment after training with vibrotactile SA. The main plot shows individual trends for the participants’ DGI scores, and the inset shows the average DGI scores. Error bars on the bar plot indicate standard error (SEM) values. DGI scores from Participant 2 are not included due to missing data. (*) indicates statistically significant changes (*p* < 0.05).

We examined the effects of six weeks of home-based balance and coordination training without vibrotactile SA on participants’ performance using the clinical outcome measures (SARA_posture&gait_, SARA, TUG, TUG-m, 5xSST, mCTSIB, and DGI.) The results of this analysis indicated that there was a statistically significant increase ($mean\;\left({post}_{-SA}-{pre}_{-SA}\right)=2.00,\;SD=1.26,F\left(1,4\right)=10.34,\;p=0.03$) in the DGI scores after six weeks of home-based balance and coordination training without vibrotactile SA ($post_{-SA}$) (Figure 6, inset), but no statistically significant differences (*p* ≥ 0.05) were found when comparing the pre- and post-training scores for the other outcome measures (Table 6). No significant main effects of group assignment or interaction effects were detected for any of the outcome measures.

Figure 5 shows the variability in the SARA_posture&gait_ score changes following six weeks of training without vibrotactile SA for both groups; while some participants (e.g., Participant 9 from Group 2) improved (by decreasing their SARA_posture&gait_ scores after six weeks of training without vibrotactile SA), others (e.g., Participant 2 from Group 1, and Participants 6 and 8 from Group 2) showed an increase in scores, and some (Participants 1 and 3 from Group 1, and Participant 10 from Group 2) maintained the same scores.

#### 3.1.3. Effects of Six Weeks of Training with Vibrotactile SA

**Figure 7:**
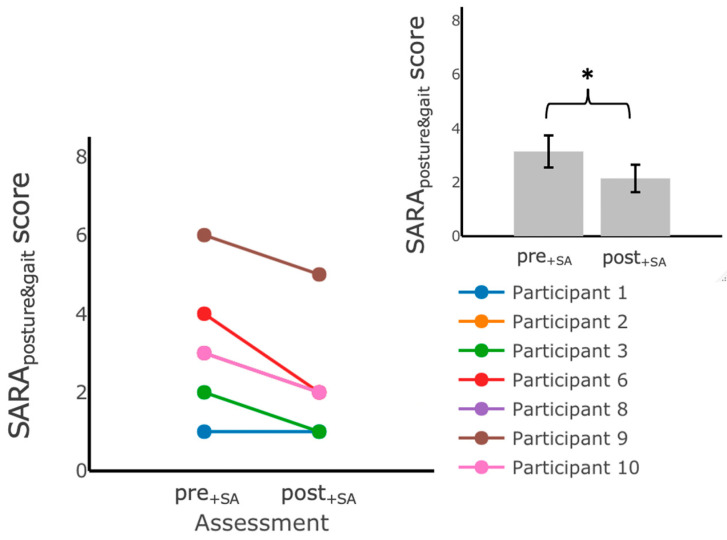
SARA_posture&gait_ scores measured during assessments performed in the laboratory before ($pre_{+SA}$) and after ($post_{+SA}$) training at home with vibrotactile SA. Participants scored significantly lower on the SARA_posture&gait_ after training with vibrotactile SA. The main plot shows individual trends for the participants’ SARA_posture&gait_ scores and the inset shows the average SARA_posture&gait_ scores. Error bars on the bar plot indicate SEM values. (*) indicates statistically significant changes (*p* < 0.05).

We examined the effects of six weeks of home-based balance and coordination training with vibrotactile SA on participants’ performance using the clinical outcome measures (SARA_posture&gait_, SARA, TUG, TUG-m, 5xSST, mCTSIB, and DGI). The results of this analysis indicated that there was a statistically significant decrease in SARA scores ($mean\;\left({post}_{+SA}-{pre}_{+SA}\right)=-1.21,\;SD=0.91,\;F\left(1,5\right)=10.67,\;p=0.02$) and SARA_posture&gait_ scores ($mean\;\left({post}_{+SA}-{pre}_{+SA}\right)=-1.00,\;SD=0.58,\;F\left(1,5\right)=22.23,\;p=0.01$) after training with vibrotactile SA (Figure 7, inset). No other statistically significant differences (*p* ≥ 0.05) were found when comparing the pre- ($pre_{+SA}$) and post- ($post_{+SA}$) training scores for the TUG, TUG-m, 5xSST, mCTSIB, and DGI (Table 6). No significant main effects of group assignment or interaction were detected for any of the outcome measures.

#### 3.1.4. Comparison of the Effects of Training with versus without Vibrotactile SA

We further examined the effects of augmenting balance and coordination training with vibrotactile SA through an analysis of the effect of intervention type (training with or without vibrotactile SA) on the change in clinical outcome measures achieved after each six-week block of training. The results from this ANOVA indicated no significant effect of intervention type on the change in outcome measures observed. We saw an estimated improvement in the SARA scores when participants trained with vibrotactile SA compared to when they trained without vibrotactile SA ($mean\;\left(\Delta_{Scores}\right)=-1.21,\;SD=1.73,\;F\left(1,5\right)=1.60,\;p=0.26)$ and the SARA_posture&gait_ scores ($mean\;\left(\Delta_{Scores}\right)=-1.29,\;SD=1.60,\;F\left(1,5\right)=3.57,\;p=0.12)$, although these did not reach the threshold for statistical significance. No significant main effects of group assignment or interaction were detected for any of the outcome measures.

### 3.2. Effects on IMU-Based Kinematic Features

**Figure 8:**
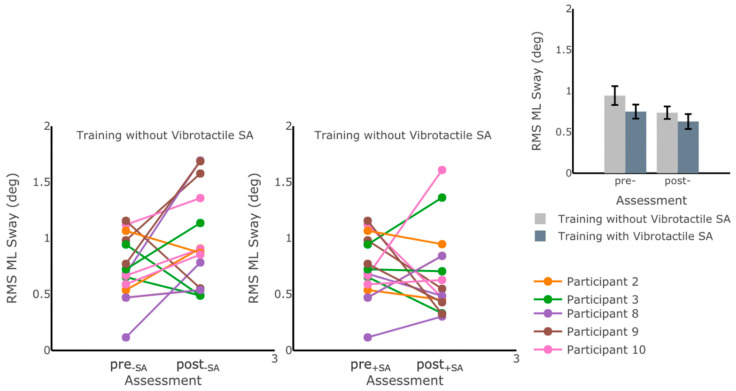
RMS ML Sway measured while participants were standing on foam with eyes open before ${pre}_{+SA},{pre}_{-SA}$ and after ${post}_{+SA},{post}_{-SA}$ each of the six-week training blocks. No statistically significant differences in postural sway measures were captured through IMU-based kinematic features (*p* > 0.05). The main plots show individual trends for the participants’ RMS ML Sway values, and the inset shows average RMS ML Sway values before and after training for each training block. Error bars on the bar plot indicate SEM values. Participants 1 and 6 were excluded due to missing data.

We also examined the effects of each training block of home-based balance and coordination training on the participants’ IMU-based kinematic features. No statistically significant differences (*p* ≥ 0.05) were found (Appendix A (Table A1)) for the IMU-based kinematic features when comparing pre- and post-training values (Figure 8) for any of the exercises performed during the assessments (Table 3).

## 4. Discussion

Balance and coordination training is currently a standard of care when treating individuals with hereditary cerebellar ataxia [14]. The goal of this preliminary study was to compare the effects of home-based balance and coordination training with and without vibrotactile SA for participants with hereditary cerebellar ataxia. 

Our results showed some evidence of the effectiveness of balance and coordination training in general. Although no statistically significant improvements were detected, the participants maintained (and showed trends of improvement in) balance and coordination outcome measures (SARA, SARA_posture&gait_, TUG, TUG-m) over the 12 weeks of training, supporting the recommendation that intensive balance and coordination training should be part of a rehabilitation program for individuals with hereditary cerebellar ataxia [13,14].

We examined the effect of the type of intervention (training with versus without vibrotactile SA) on the changes in the outcome measures achieved in each six-week block. When we compared the improvements achieved when the participants trained with vibrotactile SA to the changes achieved when the participants trained without SA, we observed a mean decrease (−1.21 points (SD = 1.60)) in the SARA_posture&gait_ score, but the difference between the interventions (training with versus without vibrotactile SA) was not statistically significant. A decreasing trend in the SARA and SARA_posture&gait_ scores was still observed in this analysis, possibly indicating that training with vibrotactile SA may provide additional benefit compared to training without vibrotactile SA, but this difference did not reach the threshold for statistical significance due to the limited statistical power of this experimental study. This finding was in agreement with a previous study by Bunn et al. [75], which reported that while the SARA and SARA_posture&gait_ scores showed similar trends of improvement in participants with hereditary cerebellar ataxia after eight weeks of training compared to a control group that did not train, statistical significance was not achieved for a minimal detectable difference in scores of 0.8 with a similarly small sample size. Bunn et al. estimated that a sample size of 64 participants or more would be needed to achieve statistical significance with a parallel study design [75]. To achieve a similar minimal detectable difference of 0.8 in SARA_posture&gait_ with our study’s 2 × 2 crossover design, we estimated that a sample size of 15 participants in total would be needed (assuming a SD = 1, α = 0.05, and power = 0.8).

Participants’ SARA and SARA_posture&gait_ scores significantly decreased following training with vibrotactile SA (i.e., assessment scores following training with vibrotactile SA compared to assessment scores immediately preceding their training with vibrotactile SA). By contrast, the participants’ SARA and SARA_posture&gait_ scores did not change significantly after training without vibrotactile SA (compared to their assessment scores immediately preceding the six weeks of training without vibrotactile SA). Therefore, only training with vibrotactile SA showed statistically significant improvements in participants’ performance on the SARA and SARA_posture&gait_ compared to their assessment scores immediately preceding that particular training block. These findings provide preliminary evidence of the potential benefits of enhancing home-based balance and coordination training with vibrotactile SA. This improvement in performance was only statistically significant for the SARA, an instrument developed specifically to capture cerebellar dysfunction, and not for the other, more nonspecific, clinical outcome measures of posture and gait performance. These findings were in alignment with previous studies investigating the effects of balance training with vibrotactile SA compared to training alone in community-dwelling healthy older adults [38,76] and individuals with unilateral vestibular disorders [39]; significant changes in performance were detected for only a subset of the performance metrics when the participants trained with vibrotactile SA compared to a control group that trained without vibrotactile SA.

The results from this study also showed a statistically significant increase in DGI scores after six weeks of training without vibrotactile SA compared to pre-training scores (i.e., immediately preceding training without vibrotactile SA), indicating an improvement in performance on gait tasks. Notably, participants did not receive vibrotactile SA during any of the gait tasks throughout either six-week training block. Therefore, it is unclear why participants did not show similar improvements in their DGI scores after completing the other six-week training block (i.e., when they received vibrotactile SA while performing static and dynamic standing tasks). Consistent with a subset of these findings, Keller et al. [26] found that participants with hereditary cerebellar ataxia improved their DGI scores and other gait-based outcome measures following six weeks of home-based gait training.

The mechanism(s) underlying improved balance and coordination performance as a result of intensive balance and coordination training in individuals with hereditary cerebellar ataxia is/are not fully understood. One possibility is that it involves better utilization of the functionally intact cerebellum; another is that other unaffected portions of the brain are recruited to improve stability [77]. The current study suggests that balance and coordination training with SA may improve cerebellar function, as captured through the SARA and SARA_posture&gait_ scores, indicating that untapped or latent cerebellar reserves could still be present in individuals with hereditary cerebellar ataxia and that cerebellar motor learning remains intact.

The analysis of the IMU-based kinematic data suggested no significant changes in any of the metrics computed, indicating that the participants’ postural sway was, on average, maintained throughout the study. A previous study from Čakrt et al. [44] reported significant decreases in CoP mean velocity and CoP 95% ellipse area during eyes-closed stance conditions after two weeks of intensive coordinative training with tongue electrotactile feedback, indicating improvements in postural stability after training. While our findings did not show similar improvements based on the IMU kinematic data, the difference in outcomes may be attributed to the difference in the IMU placement location in the two studies (on the trunk in our study and on the head in the other study), or the increased frequency of training in Čakrt et al.’s [44] study, during which participants trained twice as frequently, but for a shorter period of time (two sessions of 20 min x five days per week for a total of 20 sessions over two weeks).

The limitations of this study included the small sample size (seven study participants completed the 12-week protocol), which limited our ability to draw generalizable conclusions and obtain statistically significant results due to the limited statistical power. Larger sample sizes were challenging to obtain due to the low incidence rate of hereditary cerebellar ataxia and challenges with compliance due to the multi-week intensive study protocol. Future studies should aim to include larger numbers of participants to further assess the effects of vibrotactile SA during balance and coordination training. In addition, our study only included participants with minimal-to-moderate dependence or deficit (SARA score ≤ 14.25) [78]. Future work could include participants with more severe impairments (SARA score > 20), but additional safety considerations would need to be taken in the context of unsupervised home-based training. Furthermore, our experimental design did not include a washout period between the two training blocks.

## 5. Conclusions

This study provided preliminary evidence of the effectiveness of vibrotactile SA during home-based balance and coordination training for individuals with hereditary cerebellar ataxia. Home-based balance and coordination training with vibrotactile SA for six weeks resulted in decreased SARA and SARA_posture&gait_ scores, indicating an improvement in performance. While the findings from this study indicate some motor learning and postural stability improvements among participants with hereditary cerebellar ataxia following training with vibrotactile SA, further research with larger sample sizes is needed to assess the significance and generalizability of these findings.

## Figures and Tables

**Table 1:** Demographic information of participants. Group 1 received vibrotactile SA during the first six weeks of home-based training. Group 2 received vibrotactile SA during the second six weeks of home-based training.

Participant ID	Experimental Group	Diagnosis	Sex	Age
1	Group 1	Spinocerebellar ataxia type 2 (SCA2)	M	27
2	Group 1	Autosomal recessive cerebellar ataxia type 1 (ARCA1)	M	49
3	Group 1	Spinocerebellar ataxia type 2 (SCA2)	M	63
4	Group 1 *	Spinocerebellar ataxia type 2 (SCA2)	F	41
5	Group 1 ^†^	Friedreich’s Ataxia (FA)	F	49
6	Group 2	Spinocerebellar ataxia type 1 (SCA1)	F	32
7	Group 2 ^†^	Spinocerebellar ataxia type 1 (SCA1)	M	37
8	Group 2	Niemann–Pick C (NPC)	F	63
9	Group 2	Spinocerebellar ataxia type 1 (SCA1)	M	57
10	Group 2	Niemann–Pick C (NPC)	F	49

* Participant 4 withdrew from the study before completing the first six weeks of training. ^†^ Participants 5 and 7 withdrew from the study after completing the first six weeks of training.

**Table 2:** Participants were asked to perform home-based exercises from the following five categories during the two periods of six weeks of training.

Exercise Category	Brief Description	Vibrotactile SA Threshold
Static Standing	Standing on a firm surface, e.g., a tiled, linoleum or wood-covered floor. Participants were instructed to stand tall with eyes looking straight ahead and to minimize sway.	Anterior: 2.0° Posterior: 2.0° ML: 2.5° (on each side)
Standing on a Compliant Surface	Standing on a compliant surface, e.g., a foam pad or Bosu ball Participants were instructed to stand tall with eyes looking straight ahead and to minimize sway.	Anterior: 3.0° Posterior: 2.0° ML: 3.0° (on each side)
Arm Raises	Participants were instructed to stand tall with eyes looking straight ahead and to minimize sway as arms were lifted forward to shoulder height (90°).	Anterior: 3.0° Posterior: 3.0° ML: 3.0° (on each side)
Weight Shifting	Participants were instructed to keep feet in one position and move the body from side to side or forward and backward. The magnitude of the side-to-side or forward–backward tilt was included in the instructions (maximum tilt or medium tilt) and defined as a target position within the smartphone-based balance trainer.	Anterior: 1.0° Posterior: 1.0° ML: 1.0° (on each side)
Gait	Participants were instructed to perform gait tasks that included walking fast, walking slow, walking with horizontal or vertical head turns, side-stepping, high march, etc.	N/A (no vibrotactile SA was provided)

**Table 3:** The mCTSIB standing exercises. Three 30-second repetitions of each exercise were performed with a single IMU placed on the participants’ lower backs.

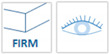	Standing with feet apart on firm ground with eyes open (Firm, EO)
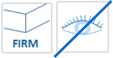	Standing with feet apart on firm ground with eyes closed (Firm, EC)
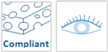	Standing with feet apart on foam with eyes open (Foam, EO)
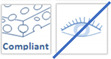	Standing with feet apart on foam with eyes closed (Foam, EC)

**Table 4:** Definitions of kinematic features extracted from the IMU sway data.

Feature	Definition	Equation
RMS ML Sway (°)	Root-mean-square of the ML angular displacement	$\mathrm{RMS}\;\mathrm{ML}=\sqrt{\frac{1}{N}\sum \theta^{2}}$ where θ=ML angle (°) *N* = number of data points
RMS AP Sway (°)	Root-mean-square of the AP angular displacement	$\mathrm{RMS}\;\mathrm{ML}=\sqrt{\frac{1}{N}\sum \phi^{2}}$ where ϕ=AP angle (°) *N* = number of data points
RMS ML Sway Velocity (°/s)	Root-mean-square of the ML angular velocity	$\mathrm{RMS}\;\mathrm{ML}\;V=\sqrt{\frac{1}{N}\sum \omega_{\theta }^{2}}$ $\mathrm{where}\;\omega_{\theta }=ML\;\mathrm{angular}\;\mathrm{velocity}\;({}^{\circ}/s)$ *N* = number of data point
RMS AP Sway Velocity (°/s)	Root-mean-square of the AP angular velocity	$\mathrm{RMS}\;\mathrm{AP}\;V=\sqrt{\frac{1}{N}\sum \omega_{\phi }^{2}}$ $\mathrm{where}\;\omega_{\phi }=\;\mathrm{AP}\;\mathrm{angular}\;\mathrm{velocity}\;({}^{\circ}/s)$ *N* = number of data points
$\mathrm{Ellipse}\;\mathrm{Area}\;({{}^{\circ}}^{2})$	95% confidence interval of an ellipse fit to angular displacement	$EA=5.99\pi \sigma_{0}\sigma_{1}\;$ $\mathrm{where}\;\sigma_{x}=\sqrt{\frac{\lambda_{x}}{N-1}}$ N=number of data points $\lambda_{x}=\;\mathrm{eigenvalue}\;\mathrm{of}\;\mathrm{matrix}\;C\;\mathrm{such}\;\mathrm{as}$ $C=\left[\begin{array}{cc} \sum \phi^{2} & \sum \phi \theta \\ \sum \phi \theta & \sum \theta^{2} \end{array}\right]$
Path Length (°)	Total angular distance traveled	$PL=\sum \sqrt{\Delta \phi^{2}+\Delta \theta^{2}}$ where Δϕ=AP angular displacement (°) Δθ=ML angular displacement (°)

**Table 5:** Participant clinical outcome measures for A1, A2, and A3 assessments. Participants in Group 1 received vibrotactile SA during the first six weeks of training (between A1 and A2). Participants in Group 2 received vibrotactile SA during the second six weeks of training (between A2 and A3).

Participant ID	Group	Asessment	SARA	SARA_posture&gait_	TUG	TUG-Motor	5XSST	mCTSIB	DGI
1	1	A1	7	1	10.8	13.6	9.1	99.1	19
		A2	6.5	1	11.6	13.8	8.3	104.6	20
		A3	5.5	1	11.8	12.7	9.0	97.9	20
2	1	A1	8	3	14.5	15.9	13.0	120	19
		A2	7	2	12.2	12.6	14.1	120	-
		A3	9.5	3	11.4	12.0	12.2	120	13
3	1	A1	6.5	2	9.5	12.6	14.0	120	20
		A2	4	1	9.6	15.1	12.0	120	19
		A3	5	1	9.0	16.5	11.4	113.6	22
5	1	A1	13	5	12.6	14.2	15.2	91.6	12
		A2	13.5	5	12.2	14.8	17.8	103.7	17
		-	-	-	-	-	-	-	-
6	2	A1	8.5	2	18.6	21.7	19.0	103.1	17
		A2	10.5	4	14.9	27.5	22.2	102.4	18
		A3	8	2	14.4	20.4	22.9	113.8	13
7	2	A1	7.5	3	11.3	22.0	12.9	100.8	21
		A2	6	2	13.0	18.0	14.5	120	17
		-	-	-	-	-	-	-	-
8	2	A1	7	2	24.6	30.1	27.1	117.9	8
		A2	7.5	3	18.1	22.7	17.0	120	10
		A3	7	2	16.5	20.9	19.3	117.3	10
9	2	A1	17	8	48.5	-	20.1	90	0
		A2	14	6	41.4	-	24.9	79	3
		A3	13.5	5	41.5	-	24.3	94.4	3
10	2	A1	11.5	3	11.2	13.3	12.1	120	18
		A2	9.5	3	12.1	13.6	11.5	120	21
		A3	8.5	2	12.4	14.0	10.8	120	22

**Table 6:** Mean (SD: standard deviation) clinical outcome measure scores at baseline and changes observed for each intervention. (*) indicates statistically significant changes (*p* < 0.05).

Outcome Measures		Baseline		Change (Post−Pre)		
		Training without Vibrotactile SA	Training with Vibrotactile SA	Training without Vibrotactile SA	Training with Vibrotactile SA	Training Overall
**SARA**	Group 1	5.83 (1.61)	7.17 (0.76)	0.83 (1.76)	−1.33 (1.04)	−0.50 (1.73)
Mean (SD)	Group 2	11.00 (4.42)	10.38 (2.72)	−0.63 (2.29)	−1.13 (0.95)	−1.75 (1.76)
	Overall	8.79 (4.27)	9.00 (2.61)	0.00 (2.06)	−1.21 (0.91) *	−1.21 (1.73)
**SARA_posture&gait_**	Group 1	1.33 (0.58)	2.00 (1.00)	0.33 (0.58)	−0.67 (0.58)	−0.33 (0.58)
Mean (SD)	Group 2	4 (2.87)	4.00 (1.14)	0.25 (1.71)	−1.25 (0.50)	−1.0 (1.41)
	Overall	2.71 (2.43)	3.14 (1.57)	0.29 (1.25)	−1.00 (0.58) *	−0.71 (1.11)
**TUG**	Group 1	11.13 (1.36)	11.6 (2.59)	−0.40 (0.53)	−0.47 (1.63)	−0.87 (2.07)
Mean (SD)	Group 2	25.73 (16.14)	21.63 (13.41)	−4.10 (3.65)	−0.43 (0.85)	−4.53 (4.15)
	Overall	19.47 (13.84)	17.33 (10.99)	−2.51 (3.26)	−0.44 (1.12)	−2.96 (3.73)
**TUG-Motor**	Group 1	13.83 (1.25)	14.03 (1.69)	−0.10 (1.32)	−0.20 (2.92)	−0.30 (3.93)
Mean (SD)	Group 2	16.28 (8.4)	21.27 (7.06)	−0.33 (6.63)	−2.13 (3.86)	−0.20 (1.01)
	Overall	17.77 (6.89)	17.65 (6.06)	−0.27 (4.28)	−1.52 (3.38)	−0.25 (2.57)
**5XSST**	Group 1	11.47 (2.94)	11.33 (3.79)	−0.60 (1.3)	−0.57 (1.56)	−1.17 (1.29)
Mean (SD)	Group 2	19.58 (6.14)	18.90 (5.92)	−0.68 (6.68)	0.43 (1.40)	−0.25 (5.63)
	Overall	16.10 (6.36)	15.66 (6.22)	−0.64 (4.78)	0.00 (1.44)	−0.64 (4.08)
**mCTSIB**	Group 1	114.87 (8.89)	113.03 (12.07)	−4.37 (3.78)	1.83 (3.18)	−2.53 (3.40)
Mean (SD)	Group 2	107.75 (14.02)	105.35 (19.43)	−2.40 (5.86)	6.03 (8.74)	3.63 (5.22)
	Overall	110.80 (11.79)	108.64 (15.94)	−3.24 (4.80)	4.23 (6.83)	0.99 (5.32)
**DGI**	Group 1	19.5 (0.71)	19.5 (0.71)	1.50 (2.12)	0.00 (1.41)	1.50 (0.71)
Mean (SD)	Group 2	10.75 (8.46)	13.00 (8.12)	2.25 (0.96)	−1.00 (2.71)	1.25 (3.59)
	Overall	13.67 (7.97)	15.17 (7.14)	2.00 (1.26) *	−0.67 (2.25)	1.33 (2.80)

## Appendix A

**Table A1:** Bar plots of IMU-based kinematic features pre- and post-training with and without vibrotactile SA. Error bars on the bar plots indicate SEM values. None of the features showed statistically significant changes for either block of training (*p* > 0.05).

	**Exercise**			
	Firm EO	Firm EC	Foam EO	Foam EC

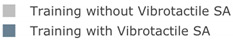
	
MS ML Sway (deg)				
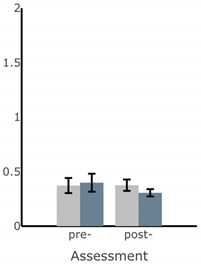
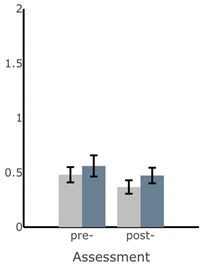
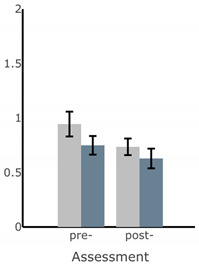
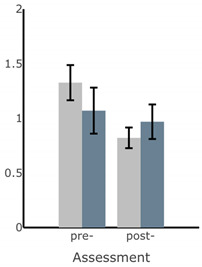
RMS AP Sway (deg)				
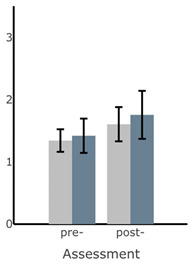
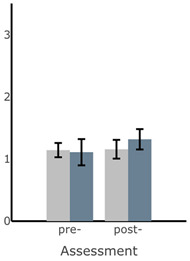
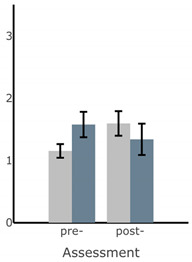
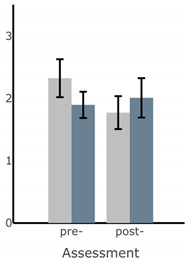
ML Sway Velocity (deg/s)				
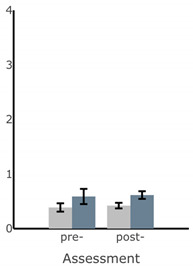
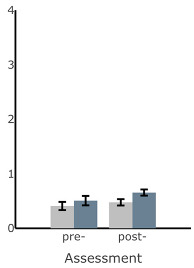
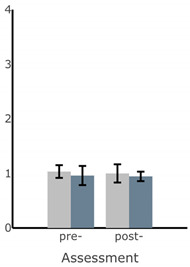
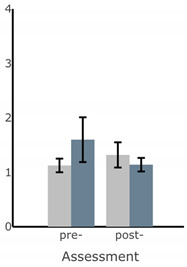
	**Exercise**			
	Firm EO	Firm EC	Foam EO	Foam EC
AP Sway Velocity (deg/s)				
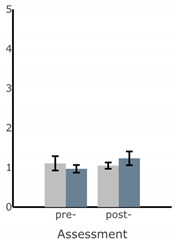
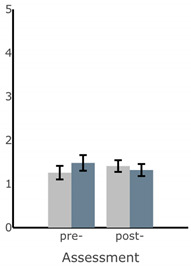
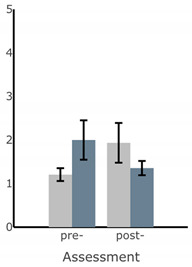
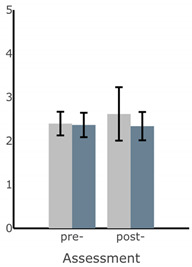
Ellipse Area of Sway (deg^2^)				
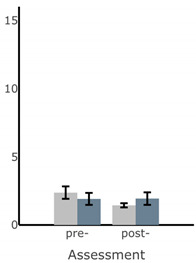
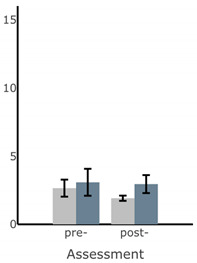
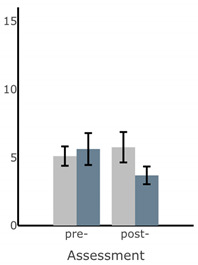
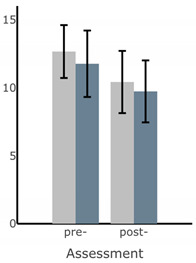
Path Length of Sway (deg)				
